# Case Report: Anti-GT1a antibody-associated ocular flutter

**DOI:** 10.3389/fimmu.2026.1684003

**Published:** 2026-02-26

**Authors:** Ye Liu, Xuejing Yan, Yu Feng, Yi Ouyang

**Affiliations:** 1 Department of Neurology, The First Hospital of China Medical University, Shenyang, Liaoning, China; 2 Department of Neurology, The First Hospital of Jilin University, Changchun, Jilin, China

**Keywords:** anti-ganglioside antibody, anti-GT1a antibody, anti-GT1a antibody-associated syndromes, Guillain-Barré syndrome (GBS) variant, ocular flutter

## Abstract

Mounting evidence suggests that antiganglioside antibodies play a crucial role in the pathogenesis of Guillain–Barré syndrome (GBS) and its variants. Among them, anti-GT1a ganglioside immunoglobulin G (IgG) antibody (anti-GT1a IgG) is most strongly associated with cranial nerve involvement. We report a rare case of a 26-year-old woman who developed ocular flutter and mild ataxia following an upper respiratory tract infection. Neurological examination revealed rapid, conjugate horizontal saccadic oscillations without intersaccadic intervals, along with a mildly ataxic gait. Brain magnetic resonance imaging (MRI) and cerebrospinal fluid analysis were unremarkable. Serological testing revealed isolated positivity for anti-GT1a IgG, with negative results for other antibodies typically associated with paraneoplastic or autoimmune diseases, such as anti-GQ1b, anti-GM1, and anti-GM2 antibodies. The patient responded well to intravenous immunoglobulin (IVIG), with marked improvement in both ocular flutter and gait disturbance. Although this presentation does not fulfill the diagnostic criteria for classic GBS, it likely represents a restricted variant within the anti-GT1a antibody spectrum. This case highlights the importance of recognizing atypical manifestations of ganglioside-associated neuropathies and supports early immunotherapy as a key factor in favorable outcomes.

## Introduction

Guillain–Barré syndrome (GBS) encompasses a spectrum of acute immune-mediated polyneuropathies, often triggered by preceding infections and characterized by varying degrees of motor, sensory, and cranial nerve involvement (1). Increasing evidence has demonstrated that antiganglioside antibodies play a crucial role in GBS and its variants (2, 3). Among them, anti-GT1a IgG antibodies have been most commonly associated with cranial nerve involvement, particularly in variants such as pharyngeal–cervical–brachial weakness (4, 5).

However, the phenotypic spectrum of anti-GT1a antibody-associated syndromes may be broader than currently appreciated. Ocular flutter, a rare abnormality of saccadic eye movement characterized by back-to-back horizontal saccades without intersaccadic intervals, is typically observed in paraneoplastic, infectious, or autoimmune brainstem syndromes and traumatic brain injury in adults (6). Its occurrence in the context of anti-GT1a antibody-associated syndromes remains infrequent and has been seldom documented in the literature.

In this report, we describe a rare case of a young female patient who exhibited isolated ocular flutter accompanied by mild ataxia. Notably, classical features of GBS, such as limb weakness and areflexia, were absent. However, the patient tested positive for anti-GT1a IgG antibodies. This case supports the hypothesis that anti-GT1a antibodies may mediate localized immune attack affecting oculomotor and cerebellar pathways and may represent a restricted variant of GBS.

## Case report

### Patient presentation

A 26-year-old woman presented with acute-onset blurred vision and dizziness. Two weeks earlier, she had experienced cough, sore throat, runny nose, and fever, which were diagnosed as an upper respiratory tract infection. She had recovered following symptomatic treatment. However, five days before admission, she developed dizziness, difficulty focusing her eyes, and unsteady walking. There was no associated weakness or numbness in her limbs, and she denied dysphagia, dysarthria, diplopia, or seizures.

Her medical and family histories were unremarkable, with no prior history of autoimmune diseases, malignancies, neurological disorders, or hereditary neurological conditions.

Neurological examination at admission revealed truncal ataxia, mild dysmetria, and an ataxic gait. Ocular motor evaluation revealed prominent ocular flutter, characterized by spontaneous, high-amplitude, rhythmic horizontal eye movements (see **Supplementary Video 1**). All other cranial nerve functions were normal. There were no abnormalities detected in limb muscle strength, deep tendon reflexes, proprioception, sensory function, or cognitive performance. Neuropsychological testing yielded no significant findings.

### Laboratory and diagnostic findings

Gadolinium-enhanced brain magnetic resonance imaging (MRI), electroencephalogram, and nerve conduction studies revealed no abnormalities. Laboratory investigations included a comprehensive panel covering complete blood counts, hepatic, renal, and thyroid function, electrolyte levels, glucose, myocardial enzyme profile, rheumatoid antibody panel, and levels of vitamin B12, folate, as well as antitreponemal and HIV antibodies. Routine blood tests demonstrated mild leukocytosis with neutrophil predominance, while inflammatory and autoimmune markers remained within normal ranges. All other results were normal. Cerebrospinal fluid (CSF) analysis indicated normal cell counts and protein levels. Serological testing revealed positivity for anti-GT1a IgG antibodies, whereas screening for other paraneoplastic, autoimmune cerebellar ataxia-related, and ganglioside-associated antibodies was negative. Detailed diagnostic results are summarized in **Supplementary Tables 1**, **2**.

### Clinical course

**Figure 1:**
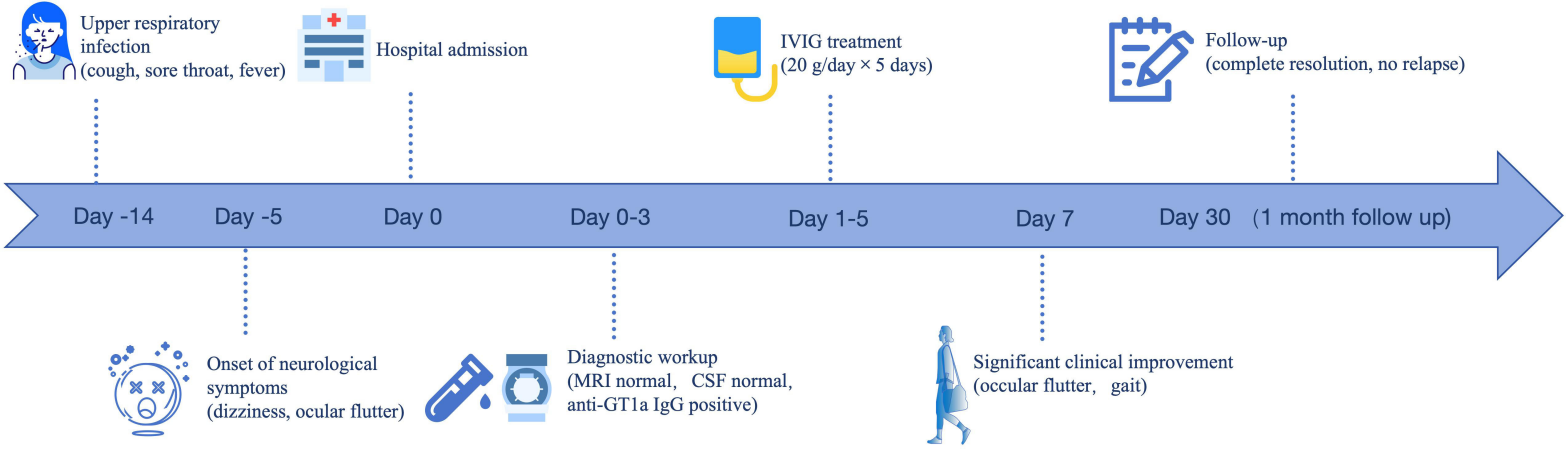
Clinical timeline of disease course and management. Timeline illustrating the clinical course, diagnostic workup, treatment, and outcomes of the patient.

Based on the patient’s medical history, clinical symptoms, and examination findings, an anti-GT1a antibody-associated syndrome is the most likely diagnosis, although it is not consistent with typical GBS. Intravenous immunoglobulin (IVIG) therapy was administered at a dosage of 20 g daily for 5 consecutive days. The patient showed significant improvement in ocular flutter and gait disturbance approximately 1 week following admission (see **Supplementary Video 2**). At the 1-month follow-up visit, the patient’s symptoms had completely resolved with no signs of relapse. A schematic timeline summarizing the clinical course, diagnostic workup, treatment, and outcome of the patient is presented in **Figure 1**.

## Discussion

This case highlights a rare and atypical presentation of anti-GT1a antibody-associated neurological dysfunction, characterized by ocular flutter and mild gait ataxia without limb weakness or areflexia. Although GBS and its variants are well recognized for their diverse clinical manifestations, ocular flutter as a primary feature associated with isolated anti-GT1a antibodies has not, to our knowledge, been previously reported.

Anti-GT1a IgG antibodies have predominantly been implicated in GBS variants involving cranial nerve involvement, including ophthalmoplegia, facial palsy, and bulbar palsy (4, 5, 7). Previous studies have shown that patients positive for anti-GT1a IgG antibodies exhibit a range of clinical presentations, often attributed to the copresence of anti-GQ1b ganglioside immunoglobulin G (IgG) antibody (anti-GQ1b IgG) IgG antibodies. Specifically, anti-GT1a IgG antibodies in conjunction with anti-GQ1b reactivity have been found in patients with oropharyngeal, neck, and shoulder weaknesses, a condition known as the pharyngeal–cervical–brachial (PCB) variant of GBS, as well as Fisher syndrome (FS), Bickerstaff’s brainstem encephalitis (BBE), acute ophthalmoparesis without ataxia, or ataxic GBS (5). However, a few studies have indicated that anti-GT1a antibodies alone can influence clinical presentation (4). For example, in a case involving a 33-year-old male patient, anti-GT1a antibodies played a causative role in the development of bilateral facial palsy with paresthesia (FDP) (8).

Reports of abnormal saccadic eye movements, including ocular flutter and opsoclonus, caused by antiganglioside antibodies are extremely rare. A recent study described a case of brainstem encephalitis presenting with ocular flutter and limb weakness, along with positive anti-GD1b IgM and anti-GT1a IgM antibodies, suggesting a potential association between antiganglioside antibodies and the occurrence of ocular flutter (9). However, in contrast to previous reports, our case has the following distinguishing characteristics: (1) only anti-GT1a IgG antibodies were present, with no other antiganglioside antibodies detected; and (2) ocular flutter was the most prominent clinical manifestation, without the typical features of GBS, FS, or BBE, such as limb weakness, oropharyngeal palsy, ophthalmoplegia, hyporeflexia or areflexia, disturbance in consciousness, or pyramidal tract signs. This case thus broadens the known clinical spectrum of anti-GT1a antibody-related syndromes.

The underlying mechanisms of ocular flutter remain a subject of ongoing investigation, with two principal hypotheses proposed: the cerebellar theory and the brainstem theory (10). The cerebellar hypothesis posits that damage or immune-mediated dysfunction of the cerebellar vermis, particularly the fastigial nucleus, disrupts inhibitory control over saccadic burst neurons, resulting in uncontrollable back-to-back saccades. The brainstem hypothesis, in contrast, emphasizes dysfunction of pontine omnipause neurons (OPNs), which normally suppress saccadic burst neurons during fixation. Loss of OPN-mediated inhibition may lead to continuous activation of burst neurons, producing high-frequency, conjugate horizontal saccadic intrusions characteristic of ocular flutter (6, 10). In our patient, the presence of isolated ocular flutter and mild ataxia, without limb weakness or ophthalmoplegia, suggests selective involvement of the neural circuits controlling saccadic inhibition and coordination. The detection of anti-GT1a IgG antibodies, which have known tropism for cranial nerves and brainstem structures (5, 11, 12), indicates that the observed phenotype may result from immune-mediated dysfunction targeting the OPNs and/or cerebellar control circuits. We hypothesize that anti-GT1a antibodies may trigger an immune-mediated disruption of inhibitory burst neuron pathways in the paramedian pontine reticular formation (PPRF) or the omnipause neuron network, leading to ocular flutter. Concurrent involvement of cerebellar outflow tracts could explain the patient’s ataxic gait. This pattern of selective vulnerability supports the concept of a restricted GBS variant.

It is crucial to consider other ocular motor abnormalities that may mimic ocular flutter. Opsoclonus, characterized by chaotic eye movements in multiple directions, is often associated with myoclonus or encephalopathy (13). In our patient, saccades were strictly horizontal, with no vertical or torsional components, and no accompanying limb myoclonus, which argues against a diagnosis of opsoclonus. Ocular clonus refers to rhythmic, slow-phase eye movements typically interspersed with fast corrective saccades, often observed in serotonin syndrome (14, 15). Our patient did not exhibit alternating slow and fast phases, making ocular clonus unlikely. Ocular neuromyotonia typically presents as prolonged tonic gaze spasms rather than rhythmic flutter. Based on brain MRI, CSF analysis, and negative results from oncological screening, potential paraneoplastic or toxic-metabolic causes—commonly associated with ocular neuromyotonia—were effectively excluded in our patient (16). Serological positivity for anti-GT1a IgG, along with a favorable response to IVIG therapy, supports an autoimmune etiology within the antiganglioside antibody syndrome spectrum.

The patient responded promptly to IVIG therapy, with significant improvement in both ocular flutter and gait. This rapid clinical response aligns with existing literature on antiganglioside antibody-mediated neuropathies, in which early immunotherapy has been shown to produce a significant therapeutic effect (17, 18).

## Conclusions

This case underscores a rare and atypical manifestation within the antiganglioside antibody syndrome spectrum, characterized by isolated ocular flutter and mild ataxia in the absence of classic features of GBS. The identification of anti-GT1a IgG antibodies and the patient’s prompt response to immunotherapy support an autoimmune, antibody-mediated mechanism targeting specific brainstem and cerebellar pathways. This case broadens the phenotypic spectrum of anti-GT1a antibody-associated disorders and suggests that localized disruption of saccadic inhibition and coordination circuits can occur independently of more widespread motor or cranial nerve involvement. Early recognition of such restricted variants is crucial for timely diagnosis and treatment, emphasizing the importance of considering antiganglioside antibody testing in patients presenting with unusual ocular motor disturbances and subtle cerebellar signs.

## Data Availability

The original contributions presented in the study are included in the article/**Supplementary Material**. Further inquiries can be directed to the corresponding author.
