# Supplementary material for: Case Report: Anti-GT1a antibody-associated ocular flutter
Source: Front Immunol. 2026 Feb 26;17:1684003. doi: 10.3389/fimmu.2026.1684003 (PMC12979508; doi:10.3389/fimmu.2026.1684003)
Supplement: Supplementary file 3 [file Table1.docx]

**Supplementary Table 1. Summary of Clinical, Laboratory, and Imaging Test Results**

| **Diagnostic findings** | **Remarkable findings** | **Reference values** |
| --- | --- | --- |
| **Neuroimaging and neuroelectrophysiology** | | |
| Gadolinium-enhanced brain MRI | No abnormal signal or enhancement | - |
| Electroencephalogram | Normal | - |
| Nerve conduction studies | Normal | - |
| **CSF analysis** | | |
| Proteins | 209 mg/L | 120-600 mg/L |
| Glucose | 3.3 mmol/L | 2.2-3.9 mmol/L |
| WBC | 2 cells/uL | (0-8) cells/uL |
| Chloride | 122 mmol/L | 120-132 mmol/L |
| Neutrophils | 50% | - |
| Mononuclear cells | 50% | - |
| **Peripheral blood tests** | | |
| WBC | 10.71×10^9^/L | 3.50-9.50×10^9^/L |
| Neutrophils | 7.94×10^9^/L | 1.80-9.50×10^9^/L |
| Lymphocytes | 2.01×10^9^/L | 1.10-3.20×10^9^/L |
| Monocytes | 0.65×10^9^/L | 0.10-0.60×10^9^/L |
| Hemoglobin | 140 g/L | 115-150 g/L |
| **Inflammatory and autoimmune markers** | | |
| C-reactive protein (CRP) | 4.6 mg/L | 0.00-6.00mg/L |
| Erythrocyte sedimentation rate (ESR) | 16 mm/h | 0-20 mm/h |
| Rheumatoid factor | 9.00 IU/mL | 0.00-14.00 IU/mL |
| Anticardiolipin antibody IgA | Negative | - |
| Anticardiolipin antibody IgG | Negative | - |
| Anticardiolipin antibody IgM | Negative | - |
| **Serum biochemistry** | | |
| Glucose | 4.37 mmol/L | 3.90-6.10 mmol/L |
| Sodium | 139 mmol/L | 137-147 mmol/L |
| Potassium | 4.22 mmol/L | 3.50-5.30 mmol/L |
| Alanine aminotransferase (ALT) | 22 U/L | 7-40 U/L |
| Aspartate aminotransferase (AST) | 17 U/L | 13-35 U/L |
| Creatinine | 68 umol/L | 41-73 umol/L |
| Urea | 4.06 mmol/L | 2.60-7.50 mmol/L |
| Lactate dehydrogenase (LDH) | 230 U/L | 120-250 U/L |
| Creatine kinase (CK) | 50 U/L | 40-200 U/L |
| **Infectious disease screening** | | |
| HIV | 0.26 S/CO | <1.00 S/CO |
| Anti-TP | 0.06 S/CO | <1.00 S/CO |
| **Vitamin status** | | |
| Vitamin B12 | 288.0 pmol/L | 145.00-637.00 pmol/L |
| Folate | 9.57 nmol/L | 8.83-60.80 nmol/L |
| **Thyroid function tests** | | |
| Free thyroxine (FT4) | 15.5800 pmmol/L | 9.01-19.05 pmmol/L |
| Free triiodothyronine (FT3) | 4.0300 pmmol/L | 2.43-6.01 pmmol/L |
| Thyroid-stimulating hormone (TSH) | 1.1915 mIU/L | 0.35-4.94 mIU/L |
| Anti-thyroid peroxidase antibody (TPOAb) | 0.8300 IU/mL | 0.00-5.61 IU/mL |
| Anti-thyroglobulin antibody (TgAb) | 0.6200 IU/mL | 0.00-4.11 IU/mL |
