# Supplementary material for: Case Report: Anti-GT1a antibody-associated ocular flutter
Source: Front Immunol. 2026 Feb 26;17:1684003. doi: 10.3389/fimmu.2026.1684003 (PMC12979508; doi:10.3389/fimmu.2026.1684003)
Supplement: Supplementary file 4 [file Table2.docx]

**Supplementary Table 2. Summary of Autoimmune Antibody Testing Results**

| **Antibody** | **Method** | **Result** |
| --- | --- | --- |
| **Autoimmune peripheral neuropathy antibody panel** | | |
| Anti-sulfatide IgG | Immunoblot | Negative |
| Anti-GM1 IgG | Immunoblot | Negative |
| Anti-GM2 IgG | Immunoblot | Negative |
| Anti-GM3 IgG | Immunoblot | Negative |
| Anti-GM4 IgG | Immunoblot | Negative |
| Anti-GD1a IgG | Immunoblot | Negative |
| Anti-GD1b IgG | Immunoblot | Negative |
| Anti-GD2 IgG | Immunoblot | Negative |
| Anti-GD3 IgG | Immunoblot | Negative |
| **Anti-GT1a IgG** | Immunoblot | **Positive** |
| Anti-GT1b IgG | Immunoblot | Negative |
| Anti-GQ1b IgG | Immunoblot | Negative |
| Anti-sulfatide IgM | Immunoblot | Negative |
| Anti-GM1 IgM | Immunoblot | Negative |
| Anti-GM2 IgM | Immunoblot | Negative |
| Anti-GM3 IgM | Immunoblot | Negative |
| Anti-GM4 IgM | Immunoblot | Negative |
| Anti-GD1a IgM | Immunoblot | Negative |
| Anti-GD1b IgM | Immunoblot | Negative |
| Anti-GD2 IgM | Immunoblot | Negative |
| Anti-GD3 IgM | Immunoblot | Negative |
| Anti-GT1a IgM | Immunoblot | Negative |
| Anti-GT1b IgM | Immunoblot | Negative |
| Anti-GQ1b IgM | Immunoblot | Negative |
| **Autoimmune cerebellar ataxia antibody panel (CBA-based assay)** | | |
| Anti-GAD65 | CBA | Negative |
| Anti-Homers | CBA | Negative |
| Anti-ARHGAP26 | CBA | Negative |
| Anti-ATP1A3 | CBA | Negative |
| Anti-CARP Ⅷ | CBA | Negative |
| Anti-NCDN | CBA | Negative |
| Anti-GluRδ2 | CBA | Negative |
| Anti-CASPR2 | CBA | Negative |
| Anti-PCA2 | CBA | Negative |
| Anti-Yo | CBA | Negative |
| Anti-mGluR1 | CBA | Negative |
| Anti-mGluR2 | CBA | Negative |
| Anti-mGluR8 | CBA | Negative |
| Anti-KLHL11 | CBA | Negative |
| Anti-Rab6A | CBA | Negative |
| Anti-Rab6B | CBA | Negative |
